# Quality of Life: A Case Report of Bullying in the Workplace

**DOI:** 10.1100/tsw.2004.13

**Published:** 2004-03-03

**Authors:** Said Shahtahmasebi

**Affiliations:** School of Mathematics and Statistics, Faculty of Health and Sciences, Christchurch Polytechnic Institute of Technology, Christchurch, New Zealand

**Keywords:** mobbing, public health, health, safety

## Abstract

The literature on bullying in the workplace describes the mental and physical ill health suffered by the victims and their families as the consequences of the bullying. The literature also discusses methods of bullying such as overt and covert physical and psychological abuse. The implications are that the consequences of abuse go far beyond the intended target; from impact on the working environment to individuals health to economic and financial loss. The literature suggests various recommendations to employers and managers to combat bullying at work. However, the common assumption within the literature has been that the bullying is done by a colleague, a line manager, or middle manager. Furthermore, it is often assumed that the executive/vice-chancellor, human resources, the trustees, or the governing board are unaware of bullying in their workplace. In this article, it is argued that cases of bullying (whether due to isolated individuals, competition, rivalry, power, or pure meanness as is reported in the literature) can only thrive in a bullying management culture. Therefore, debate and policy formulation must be directed at government level in the first instance. The case report is intended to raise some relevant issues to stimulate a debate and more research in this area.

## INTRODUCTION

Much has been written about the consequences of bullying as a social problem both in school and the workplace. The literature covers the various aspects of bullying, e.g., theoretical considerations[1]; management environment and gender[2]; psychological violence, misplaced loyalty, and methods of bullying (e.g., overt [visible physical and mental abuse] and covert [subtle abuse])[3]; the effects of bullying[4,5,6,7]; bullying at school[8,9,10,11]; and causes of bullying[12,13]. MaAvoy and Murtagh[14] provided a brief description of workplace bullying. An example of overt and covert abuse by an employer is demonstrated in a case narrative[15]. Various authors make recommendations to the employers in order to combat bullying in their work environment[16]. Glendinning[17] suggested that it is the role of human resources (HR) to deal with bullying and administer relevant policies in the workplace, which often has tremendous implications for the organisation.

Most studies of bullying have concentrated on the victims' characteristics. Very few people ever victimise themselves; to have a victim, there must be a seeker[18]. Human interaction is a process and therefore is governed by dynamic codes of conduct. Some individuals, for whatever reasons, breach these codes to inflict suffering on others with whom they are bound by and share a common goal, through the organisational policy objectives and contracts. Very few studies have paid attention to the characteristics of the organisations, the bullies, or the environments where bullies thrive. An important question to ask is: What governs these individuals' behaviour? Without a proper study design to treat bullying as a process and to include the dynamics of human behaviour, our understanding of bullying behaviour could be vague and confused and at best one-sided. For example, Einarsen[12], in an attempt to address the nature and causes of bullying, on the one hand reported that the victims of harassment were more oversensitive, suspicious, or angry with low self-esteem or anxiety. On the other hand, Einarsen[12] argued that the personality of the victim may provoke aggression in others. Einarsen[12] quoted competition, envy, and the aggressor's own self-doubt as three main reasons for bullying. However, these results were based on a subjective survey of the victims. The author, quite correctly, reported that without longitudinal studies, personality factors may well be the outcome of bullying. It is not surprising then that assertiveness in the workplace is advised[19]. Indeed, most employers, through their HR division, offer staff self-development/assertiveness courses.

There are a number of problems with this strategy. There are no clearly defined boundaries for when assertiveness becomes aggression and bullying. What good will assertiveness or any antiharassment policy do in a working environment and culture based on fear amongst staff, rumours, lies, falsifying of facts, misinformation, and so on, often referred to as predatory bullying[12]? And what are the implications of implementing recommendations from poorly designed studies and lack of real evidence? For example, Manthei's[20] comparison of methods of teaching assertiveness was flawed and lacked academic rigour; it appeared to breach ethics regulations and was a badly designed and poorly conducted study with an inappropriate methodology that does not relate to the aims of the study. The author's conclusions appeared to be her own views and do not relate to the results. Therefore, policies developed based on such studies will create more problems for the organisations rather than resolving them.

For practical purposes, perhaps, the brief discussion by McAvoy and Murtagh[14] provided a more comprehensive collection of issues from the prevalence of morbidity in the individual victims to the morbidity of the workplace environment. They also reported that apart from the negative effects on physical and mental well being, bullying can have negative cognitive effects, e.g., lack of initiatives. Such practices will not be without economic and other social consequences. For example, McAvoy and Murtagh[14] reported that statistics from the U.K. national workplace bullying advice line suggest 20% of the cases are from the education sector, 12% from healthcare, followed closely by 10% from social services. It is not surprising therefore that the associated costs of bullying in the U.K. alone is estimated at £2–30bn per annum. Similarly, a Canadian report[21] suggested a cost to employers of $3.5bn and when the costs of wage replacement, health, and medical care were included, the costs were estimated at $16bn. See also Sheehan for the Australian experience[22].

Bullying and harassment, though studied as one phenomenon, take different forms and shape. However, it is argued that bullying thrives in an environment where the organisation has a bullying culture[4,12]. Examples of harassment may include belittling opinion, public humiliation, withholding of access to training, unnecessary disruption, obstruction, threats of dismissal, shifting of goalposts, removal of responsibility, and undue pressure to produce work[4]. Sheehan[22] reported psychological abuse in the workplace and discussed the damage caused, and the legal and economic risks to organisations that failed to address the problems.

## CASE REPORT

Unsurprisingly, the case report concerns a tertiary education institution. The subject has kindly given permission to outline some of the main events representing unprofessional behaviour by the institution. In order to maintain anonymity, details of events have been kept to a minimum.

The subject accepted an offer of a post from an educational establishment. The subject was brought in with the specific mandate to carry out research and development (R&D) consultancy with a small teaching component. The advertisement and the bumph gave a positive picture of the institution using phrases such as a highly supportive, friendly, and dynamic working environment; teaching staff expert in their fields; and high-quality research and researchers with international standing. A quick Internet search established that the claims about international standing was standard hype and by “dynamic” it meant a high staff turnover. The subject’s final choice over other job offers was influenced by the challenges of the post.

One of the subject’s mandates was to identify problem areas and report them to the management. Initially, things got off to a flying start and despite the negative rumours about the institution and the management, a number of initiatives got underway. These initiatives proved popular with staff. Within the first few weeks of starting work, the subject realised that the institution had huge problems in terms of culture, management style, staff, and above all vision. All the management posts had been filled via internal promotions despite attracting credible and well-qualified external candidates and against the wishes of staff members who participated in the selection process. Most of the managers/staff had been in the same institution for many years. The existence of tribalism suggested the divide-and-rule style of management. Access to resources in particular to do research is the privilege of those belonging to the right tribe. Symbolism appears the central driving force in management style and staff perceptions of status. For example, office size, position, and job title (e.g., team leader) signified academic status as opposed to experience, qualification, and performance. The institution was administered by staff without relevant management experience (in particular people skills) and qualifications. These internal appointments were apparently made after open competition with staff competing with credible, well-qualified, and highly experienced candidates. Clearly, such a management style was a recipe for disaster, particularly where new blood was recruited to carry out the education developmental work and delivery. The subject reported that the frustration of experienced and qualified staff working under such management had soon given way to friction and confrontation, which quickly turned to hostility, abuse, and harassment.

The subject made a report to the management based on the above observations and suggested that for the institution to achieve its goals, a more supportive management practice was essential. In the report, the subject further suggested that change in practice and recruitment of new blood at senior level to be a necessity. Moreover, the report recommended that as a first step, the institution needed to consider a retraining programme for its current managers. With hindsight, the management did not want to see that kind of report! What followed next was a long (nearly 3 years) sustained campaign against the subject. The initiatives one by one (without justification and in breach of the institution’s own policies) were cancelled, the subject was excluded from input and contribution, workload was increased, and rumours about the subject were spread. Consultations with HR and their promise of resolving the situation were not only fruitless, but also led to further campaigns. Included in the arguments with the management was the treatment of staff. However, the subject underestimated the fear amongst the staff; they were unwilling to demand their own rights and were prepared to be passive bystanders grateful for having a job. This summarised the workplace culture of the institution. Managers with no track record and experience in R&D suddenly became experts and criticised the subject. Needless to say, the subject was quick and efficient in dismissing such criticism, which appeared to make the matters worse and help the campaign against the subject to intensify. Delaying tactics and obstruction also included intrusion, not processing forms until after the closing date, access to personal files/materials without the knowledge or permission of the subject, interfering with processes, and contacting external agencies with the specific view of obstructing the subjects progress. The subject reported these as the institution's immoral activities and malpractice by design.

The subject wondered: If management was so critical, why would they not call a meeting in order to terminate her contract? Not surprisingly, some material and statistics became available through the unions that revealed an unwritten "no termination of contracts" policy for fear of legal repercussion. The senior management including HR had a history of aggressive behaviour and harassment against staff, were engaged in spreading rumours, setting up colleagues against colleagues, obstruction, positive support for the subject from other heads of department being falsified as negative, and threatening behaviour. Instead of following complaint procedures, the management met every complaint with a counter complaint and the use of their external social contacts to obstruct. Also revealed were consistent complaints against the same managers and pattern of behaviour by the institution (similarly using humiliation, threats, undermining, belittling, etc.): the victims were either silenced, had to leave their posts, or on long-term sick leave whilst the offending managers received promotion! In fact, during a face-to-face conversation about the structure of the institution and procedures with the head of department, the subject was told that the head was the real decision maker, indicating that there was no point contacting even the executive/vice-chancellor.

The subject was a confident and professional individual with a capacity to articulate and debate a point of view with evidence. The irony was that the employer consistently used this virtue negatively against the subject, first covertly and then overtly in the statements of complaints. Examples of false assertion by the managers included that staff found the subject aggressive, intimidating, and put the institution at risk!

The consequences of working in such an environment were multidimensional. First of all, the effects on the subject's personal life can be categorised as individual and family. The individual effects include health, financial, career, and family problems: the subject suffered mental and emotional stresses manifesting as physical and emotional symptoms. Quite apart from the emotional suffering, the combined effect of ill health was debilitating, led to a loss of confidence, reduced output, and financial loss in fees for GP/consultant/psychiatrist/medicine. The effect on the subject's family was most stressful. The family suffered severe emotional stress and ill health, which strained the relationship between the subject and the rest of the family, leading to separation.

For the employer, it was the loss of an active dedicated employee who put institutional goals ahead of personal objectives. Lost productivity. Lost progress. Lost market. Therefore, lost long-term investment, possible lost returns, and unnecessary expenditure in lawsuit(s) that could have been invested in the institution for its staff and students' future.

## DISCUSSION

Recommendations to combat bullying in the workplace are directed at the employer[16,23], e.g., employers must educate their line managers and should investigate claims thoroughly. By the end of the last century, most western governments acknowledged bullying and its consequences as a major problem, and issued policy statements that hold the employers responsible for managing a stress-free working environment. This is no different than the executive/vice-chancellor's passing of the problems to the source of the problem to resolve! It is not surprising, therefore, that the number of cases *after* government legislation for stress-free working environment, including this one (also see[15,22,23,24]), demonstrated that the offending employers continued to show a disregard for the law, let alone any implementation of antiharassment recommendations. The politicians, trustees, auditors, legal community, staff, etc. all know who the offending organisations were — the question is: Why do we allow bullying behaviour to continue?

Clearly, it is not as easy to study perpetrators or organisations. Any references to the bully or organisation characteristics are usually obtained from the victims' accounts. Most studies also rely on surveys of victims or legal/court case notes. However, very few cases make it to the courts and a majority accept out-of-court settlements (for obvious reasons, e.g., low levels of self-esteem, stressed and depressed, ill health). However, the settlement often comes with a gag order that prevents the victim from discussing any aspects of the case with anyone! Therefore, we know very little about the victims or the effect on their families; the untold damage to health and mental well being, in particular the emotional well being of the children. In addition, our information on bullying is collected after the event, after a period of sustained bullying and stressful working environment. Thus, although the data set will have contained historical information, it is essentially cross-sectional information on an individual exhibiting low self-esteem, uninterested, depressed, and unable to articulate. It is not surprising that some authors recommend self-development training (e.g., assertiveness) models to combat bullying! This type of model accepts bullying in the workplace and places the onus on the employee to prepare for it.

In attempting to understand why certain senior managers, who already hold positions of power and have established “empires”, behave in such a manner in today’s society, the subject reported that almost all the comments received from other colleagues pointed to the insecurity and low confidence of the management due to their lack of experience. These comments appear in line with the literature quoted in the introduction. However, they do not explain the treatment of other teaching staff, who did not pose a threat to anyone and simply wanted to do their jobs. One simple explanation could come from the literature. Such a management style is reminiscent of schoolyard/playground bullying. There is some evidence to suggest school bullies suffer from psychological and mental health problems, which go undiagnosed and untreated[10,11], furthermore, school bullying may be transferred to adulthood and the workplace[8]. On the other hand, legal risks may have influenced management behaviour. The literature provides examples of the legal precedent of employees being awarded damages against employers who failed to provide due care and support for appointing inexperienced staff into management roles[22].

An important issue arising from the case report and which did not feature in the literature was the effects on the victim’s family quality of life: (1) sudden change in the family, (2) sustained strained family relationship and stress, and (3) the workforce as passive observers.

It is commendable to see McAvoy and Murtagh[14] suggest that the medical profession ought to take a serious role to prevent bullying in the workplace. I would argue further that this role has to be in conjunction with other actors — we must adopt a holistic approach such as the Good Life Project (http://lists.cpit.ac.nz/thegoodlife/). The approach should take account of the recent history; unmonitored changes in national and international politics, economic, welfare, workers unions, and social policies, and their possible effects on the society — in general the social shift in the context of globalisation[25,26] Only a concerted multidisciplinary-led attempt to include members from the judiciary, academia, government, employers, unions, health sector, etc., will have some credibility.

## BIOSKETCH

**Said Shahtahmasebi** is currently research methodology consultant and statistician at Christchurch Polytechnic Institute of Technology, Christchurch, New Zealand. His work experiences are mainly in the United Kingdom and cover a number of fields including public health, mental health, nursing, operational research, food, nutrition. and gerontology. His area of interests and expertise are related to longitudinal modelling of health related behaviour. He has set up the Good Life Project (http://lists.cpit.ac.nz/thegoodlife/) in New Zealand, which attempts to investigate food holistically.
